# Minimizing ice contamination during specimen preparation for cryo-soft X-ray tomography and cryo-electron tomography

**DOI:** 10.1016/j.yjsbx.2024.100113

**Published:** 2024-10-18

**Authors:** Chia-Chun Hsieh, Zi-Jing Lin, Lee-Jene Lai

**Affiliations:** Experimental Division, Synchrotron Radiation Research Center, 101 Hsin-Ann Road, Hsinchu Science Park, Hsinchu 300092, Taiwan, ROC

**Keywords:** Cryo-sample preparation, Ice contamination, Grid box, Cryo-soft X-ray tomography, Cryo-electron tomography

## Abstract

Cryo-soft X-ray tomography (cryo-SXT) is a newly developed technique for imaging 3D whole cells in nearly native states. Cryo-SXT users require the preparation of numerous cryo-sample grids to use the allocated beamtime to study cellular phenomena under various conditions. Therefore, it is important to promptly prepare cryo-sample grids as efficiently and carefully as possible to minimize ice contamination on the frozen sample grid. In this study, we designed a cryo-multi-grid-box storage system, which includes a shell, funnel holder, and multi-grid-box container. Our system not only increases the number of cryo-sample grids that can be temporarily stored but also reduces the frequency of cryo grid-box container transfers, thus decreasing the probability of forming ice on the grid. We have also applied this system to A549 cryo cell grid preparation. The correlative images from cryo-light microscopy and cryo-SXT showed that limited ice had formed on the grid when preparation was performed using our system. Additionally, 3D images of mitochondria with the lamellar shape of the cristae could be observed in our cryo-SXT results. Our cryo-multi-grid-box storage system can be used for cryo-SXT and cryo-electron tomography (cryo-ET) applications.

## Graphical abstract

Simultaneously preparetriple cryo-sample gridsfor a transfer in one-time to reduce ice contamination on the sample grids for cryo-SXT and cryo-ET imaging.

**Figure:**
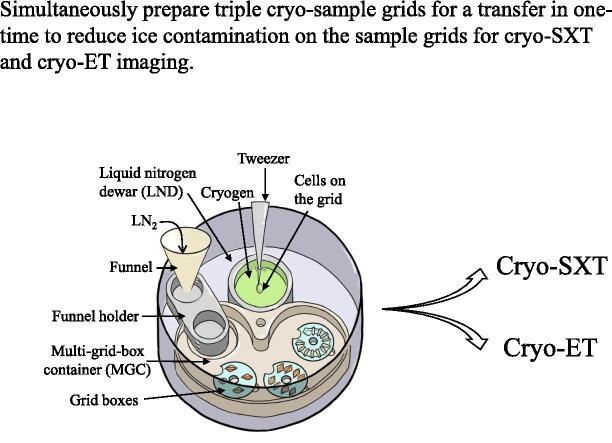

## Introduction

Cryo-soft X-ray tomography (cryo-SXT) is an emerging technology to image the three-dimensional (3D) morphology of whole cells (Larabell and Nugent, 2010, Duke et al., 2014, Varsano, 2016). In the water window range, the penetration depth of cryo-SXT for bio-specimen is about 10 μm, allowing for imaging of the hydrated biological sample without the need for sample sectioning. Furthermore, cryo-SXT can be performed in natural contrast without needing a contrast agent or dye labeling (Kirz et al., 1995, Schneider et al., 2012). Thus, cryo-SXT has become an essential tool for bridging the gap between cryo-electron microscopy (cryo-EM) and light microscopy (Chen et al., 2016).

Bio-samples are susceptible to damage from soft X-ray exposure; it is necessary to keep them frozen to avoid radiation damage. Generally, cryo bio-samples can be mounted into one of two types of sample holders: they may be pipetted into a very thin capillary tube (Le Gros et al., 2005), or a gold grid is applied as a sample supporter to which the cells are seeded on a long particular grid (Hagen et al., 2012) or on a standard 3.05 mm diameter electron microscopy grid (Conesa et al., 2016).

Users are often required to prepare numerous cryo-samples under various cell conditions simultaneously to allow a direct comparison of cell phenomena. Before seeding the cells on the grids, the carbon side of the grid has to be glow-discharged to form a hydrophilic surface to help cell adhesion. Cell seeding and incubation may take 8 ∼ 48 h, depending on the conditions. After incubation, the grid with cells is blotted with filter paper to absorb excess water. The sample is quickly dipped into a cryogen, such as liquid ethane or liquid propane,that has been pre-cooled by liquid nitrogen (LN_2_) to avoid ice crystal formation inside the cells (Hsieh et al., 2023, Groen et al., 2021). However, ice contamination can form on a sample grid or grid-box container (GC) during extended preparation time or during the transfer of the GC, whose function is to store the cryo-sample grids temporarily during cryo-sample grid preparation. Once the GC is complete, it has to be transferred to relocate the cryo-sample grids, which creates another opportunity for ice to form. Ice formation can increase the thickness of the sample, thereby reducing the photon flux of cryo-sample absorption, resulting in low-quality cryo-SXT images. Therefore, it is important to complete cryo-sample grid preparation as soon as possible and transfer them in one-time.

The space inside a small liquid nitrogen dewar (LND) is limited for plunge freezing machines that contain a secondary cryogen temperature controller, such as Leica EM GP2. Typically, GCs in these machines only accommodate one grid box, which holds four bare grids in a standard grid box or 12 grids in a specialized grid box. Once the grid box is fully occupied with the sample grids, the GC must be transferred to relocate the sample grids. Therefore, a general GC may not meet the demand of laboratories to prepare many cryo-samples simultaneously, especially for users in cryo-SXT. In this study, we designed a multi-grid-box storage system with a modified container. The container possesses a space with three concave grooves that can be loaded with three grid boxes. This system allows users to prepare triple cryo-sample grids and store those sample grids in a modified container for one-time transfer to reduce ice contamination on the sample grids. Our system is also helpful in cryo-sample preparation for the cryo-ET community.

## Results

### Design of a cryo-multi-grid-box storage system

**Fig. 1:**
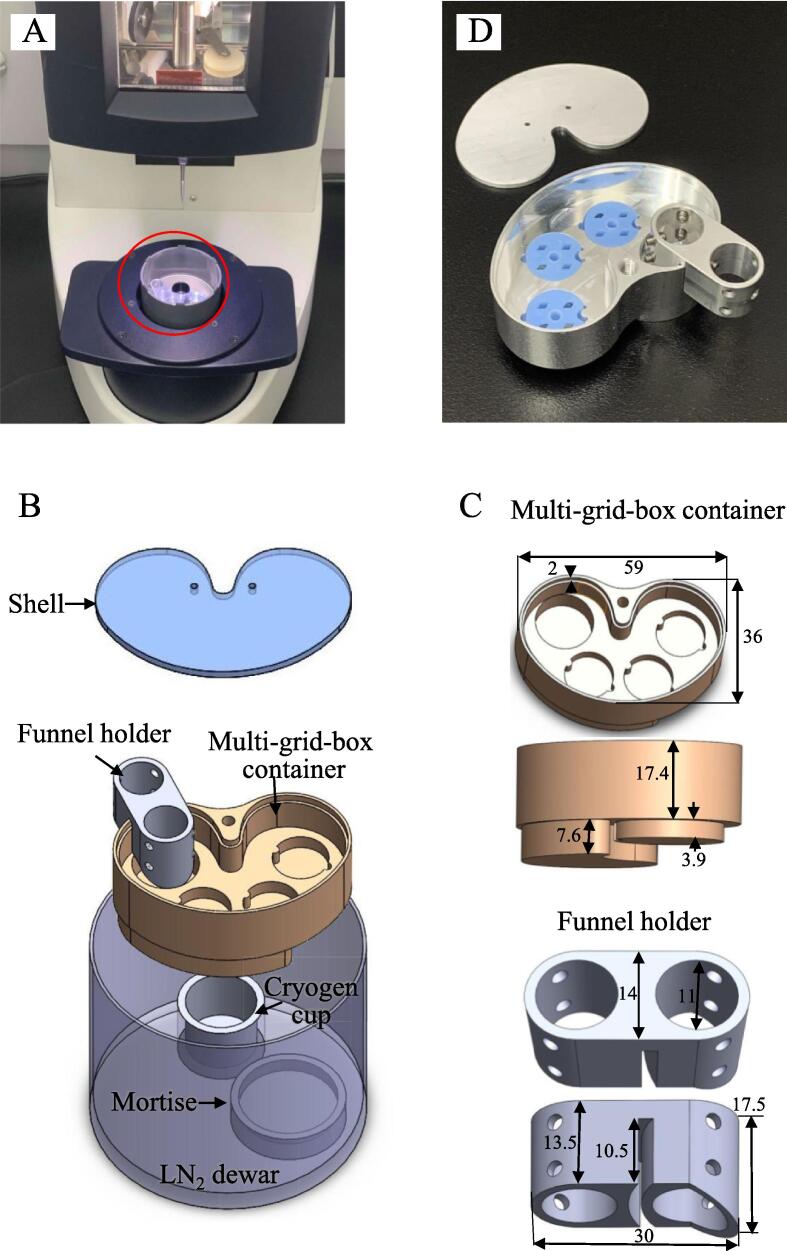
Cryo-multi-grid-box system. (A) A photo of cryo-multi-grid-box system (red circle) in a plunge freezer machine. (B) A drawing of each component of the cryo-multi-grid-box system: shell, funnel holder, and multi-grid-box container. The cryogen cup, LN_2_ dewar, and mortise are the existing components in the freezer machine. (C) Detailed 3D illustration of the multi-grid-box container in top and side views (up), and funnel holder in top and side views (down). (D) A photo of the assembled cryo-multi-grid-box system. Dimension, mm. (For interpretation of the references to colour in this figure legend, the reader is referred to the web version of this article.)

We designed a cryo-multi-grid-box storage system that is compatible with the small LNDs found in a standard plunge freezing machine (Fig. 1A). This storage system consists of three components: a shell, funnel holder, and multi-grid-box container (MGC). Each component has its unique function and specific dimensions to fit existing LND components (Fig. 1B).

A shell serves as the lid of the MGC, preventing contact of the prepared cryo-sample with moisture from the air during the transfer of the MGC. Two small holes are incorporated into the shell designed to allow manipulation with tweezes and to allow nitrogen gas release. Fig. 1C displays the detailed dimensions of the MGC and funnel holder from the top and side views. The MGC was designed in a heart shape with dimensions of 59 x 36 x 17.4 mm^3^ (W x L x H) to fit an existing secondary cryogen component located inside a standard LND. A 2 mm deep groove around the top of the MGC was designed to secure the shell. Inside the MGC, we designed four grooves on the bottom, including one deep groove on the left side to prevent LN_2_ splashing while pouring through the funnel and three shallow grooves on the right side for loading grid boxes. Each shallow groove has small concave and convex holes on the opposite sides of its edge. The convex hole serves as a locker to lock a standard round grid box and to prevent the rotation of the grid box, and the concave hole works as a space buffer to allow tweeze to remove a grid box from the MGC. Therefore, in the process of cryo-sample grid preparation, cryo-sample grids can be sequentially and temporarily stored in the three grid boxes loaded on the shallow grooves of the MGC. In the upper middle part of the MGC, a tap hole is included to allow the MGC to be removed from the LND using a handling rod or general long screw. This makes it possible to prepare numerous cryo-sample grids and place them in the MGC. A single transfer of the MGC can then be achieved to further cryo-fluorescence screening or to a traditional dewar for long-term storage. The side view of the MGC reveals two blocks with different shapes and heights of 7.6 and 3.9 mm on the bottom, designed to fit an existing mortise in the LND. Because of the limited space of the LND, LN_2_ is possible to rush the sample-grids and splash into the cryogen once pouring the LN_2_ into the LND with a thermos cup. Therefore, we designed a funnel holder to support the funnel and guide the LN_2_ flow. The elliptically shaped holder includes two caverns with diameters of around 11 mm for fitting a standard small funnel pipe, with two different heights visible from the side view. On the bottom of the holder, a deep groove in the center allows the holder to be mounted onto the wall of the MGC, which can separate the outer and inner caverns corresponding to the outside and inside the MGC. After the entire assembly of the system, the bottom of the holder in the outer cavern side is lower than the top of the cryogen cup, allowing LN_2_ to be filled quickly through the funnel into the LND to cool the cryogen and MGC without splashing, keeping the temperature of the cryogen stable. Furthermore, the depth of the holder in the inner cavern side makes the bottom of the holder lower than the top of the groove, which allows LN_2_ to be filled through the funnel into MGC quickly without rushing to the cryo-sample grids. Moreover, we designed four tap holes in each cavern, which allow adjustment of the tightness between the funnel pipe and cavern using screws to prevent tilt or movement of the funnel, thereby making LN_2_ flow smoothly into either the MGC or LND. All components of the multi-grid-box system are fabricated with aluminum materials, as shown in Fig. 1D.

### Handling of the cryo-multi-grid-box storage system

**Fig. 2:**
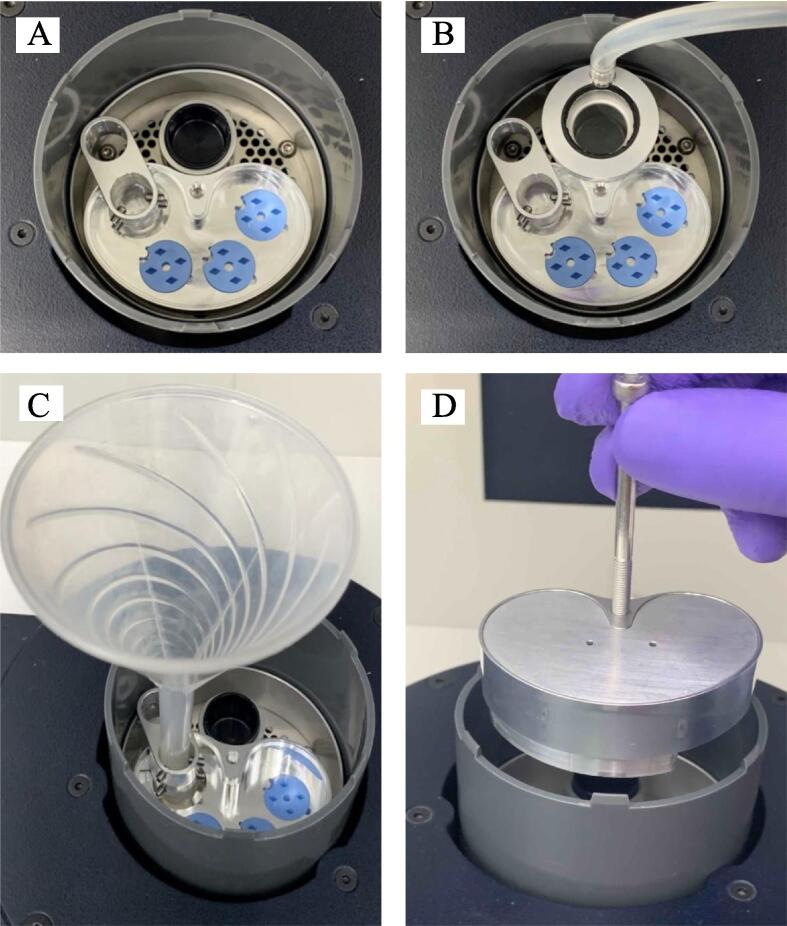
Photos of the operation of the cryo-multi-grid-box system. (A) Assembled funnel holder and three grid boxes in the cryo multi-grid-box container inside the LN_2_ dewar. (B) Lid of the cryogen cup connection with rubber pipe for cryogen gas. (C) A funnel mounting on the funnel holder for passing LN_2_ to either the cryo multi-grid-box container or LN_2_ dewar. (D) Removing the shell covered cryo multi-grid-box container from a LN_2_ dewar using a general long screw.

Fig. 2 displays the handling of the cryo-multi-grid-box system in the small LND. Several preparatory steps are required prior to cryo-sample preparation. First, three grid boxes are loaded inside the MGC, and the funnel holder is mounted on the wall of the MGC. All components are loaded into the LND (Fig. 2A). Second, LN_2_ is poured into the LND to pre-cool a secondary cryogen cup. After the system has sufficiently cooled, cryogen gas is passed through a pipe connected to the lid of the cryogen cup to fill into the pre-cooled cryogen cup, where the gas is condensed to liquid-phase cryogen (Fig. 2B). Third, the cryo-sample is prepared by quickly plunging the sample grid into the liquid cryogen. Once these steps are completed, the prepared cryo-sample on the grid can be moved to the grid box in the MGC. Sample preparation can continue until the sample grids fully occupy the three grid boxes. The MGC can be transferred. During many cryo-sample grids preparation, it may be necessary to replenish LN_2_ through the funnel on different caverns of the funnel holder to either the MGC or LND to continue cooling the system (Fig. 2C). Once preparation of the cryo-sample grids is complete, the funnel holder can be removed from the MGC using tweezers. The MGC can be covered with the shell to reduce ice contamination caused by contact with moisture in the air during the transfer process. Finally, a long screw can be used by locking a tap hole on the top middle of the MGC to remove it from the LND for further processing (Fig. 2D; Supplementary video S1).

### Characterization of the cryo-samples with light microscopy and soft X-ray tomography

**Fig. 3:**
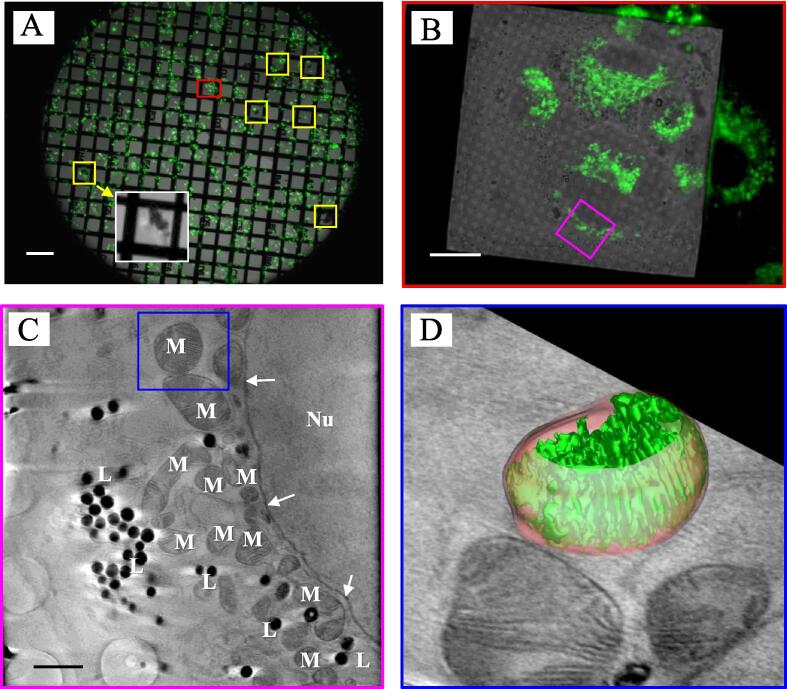
Images of cryo A549 cells on a standard-sized gold grid from cryo-light microscopy and cryo-soft X-ray tomography. (A) A merged bright-field and fluorescence image of frozen A549 cells on the grid using a 5x lens. The inset (white square) shows the magnified image of ice with cells seen by bright-field microscopy. Location of ice: yellow squares. Scale bar: 200 µm. (B) A merged bright-field and fluorescence image of frozen A549 cells on the grid using a 100x lens from the area marked by a red rectangle in (A). Scale bar, 20 µm. (C) Virtual slice image of a reconstructed soft X-ray tomographic Z-stack from the region of interest marked as a pink square in (B). Nucleus: Nu. Nuclear membrane: white arrows. Mitochondria: M. Lipid droplets: L. Scale bar, 2 µm (D) Segmented 3D image of a mitochondrion from a blue square area in (C). Outer (orange) and inner (green) mitochondrial membranes. (For interpretation of the references to colour in this figure legend, the reader is referred to the web version of this article.)

The quality of frozen cellular specimens on grids prepared using the designed cryo-multi-grid-box storage system was examined by cryo-light microscopy (Fig. 3A, B). The A549 cells were stained with MitoTracker™ Green FM before freezing. The results showed large ice aggregations as dark regions on the grid with cryo-bright-field light microscopy. Thus, we merged images of bright-field and fluorescence microscopies to observe the relative location of cells and ice contamination. The merged image also allowed us to identify regions of interest (ROI) for cryo-SXT imaging, as shown in the pink square of Fig. 3B. We estimate that the coverage of ice contamination on the Au grid surface is less than 1 %. Therefore, the SXT image of the sample is unaffected by ice contamination if the location of ice contamination is away from the region of interest (ROI). However, if the location of ice contamination is close to the ROI when the sample is rotated at a high tilt angle, the ice increases the thickness of the sample. It reduces the penetration depth of the soft X-ray into the sample, resulting in a decrease in the SXT image quality of the sample. A virtual slice image from the reconstructed Z-stack clearly shows organelles, such as nuclear membranes, mitochondria, and lipid droplets (Fig. 3C). The inner layer of cristae in the mitochondria is also clearly observed. The inner and outer mitochondrial membranes can be visualized using image segmentation (Fig. 3D). Ice was not observed in the cryo-SXT image. The results revealed that only a small amount of ice aggregated near the cells on the cryo-sample grid prepared using the designed MGC, which we observed from images generated by bright-field and fluorescence microscopies.

## Conclusion

In this work, we designed a cryo-multi-grid-box storage system consisting of several components, including a shell, funnel holder, and MGC. The aim was to allow cryo-SXT users to prepare multiple frozen cellular specimens on grids with less ice contamination. Our designed MGC can store three times the number of cryo-sample grids than a conventional GC with one grid box. Moreover, transferring triple the number of prepared cryo-sample grids simultaneously not only reduces the transfer frequency and saves time but also declines ice contamination on the container during the transfer process. We applied imaging tools of cryo-light microscopy and cryo-SXT to characterize ice contamination on the cryo-sample grids that were prepared in our system. The results reveal that only a tiny amount of ice contamination was observed on the cryo-sample grids. Our designed system can be applied to a standard plunge freezing machine with a crowed space of LND. However, this system could potentially be modified and customized to fit different plunge freezing machines to further increase the number of cryo-samples that can be prepared simultaneously. Moreover, the system can be utilized by both cryo-SXT and cryo-EM communities for cryo-sample preparation.

## Materials and methods

### Cell culture and reagent

The human lung carcinoma cell line (A549) was purchased from Bioresource Collection and Research Center in Taiwan and grown in Ham's F12K medium, supplemented with 10 % fetal bovine serum (FBS) and 1 % penicillin–streptomycin. Cells were cultured at 37 °C in 5 % CO_2_. MitoTracker Green FM was purchased from Thermofisher/Invitrogen.

### Sample preparation for cryo-light microscopy and cryo-soft X-ray tomography

A549 cells were grown overnight on standard gold grids coated with holy-carbon films (QUANTIFOIL R 2/2 on Au G200F1 finder grids, Quantifoil Micro Tools GmbH). The A549 cells were stained with MitoTracker Green FM to highlight mitochondria. Before freezing the cells, the sample grids were placed in the Leica EM GP freezing system and 100 nm gold particles (BBI) were added. After 3 sec of blotting to remove excess water, the grids were quickly dropped into the liquid ethane in the freezing system. The plunge frozen samples were removed into our MGC storage system for further sample screening using a cryo-fluorescence system assembled with cryo-stage (CMS196, Linkam Scientific Instruments Ltd.) and fluorescence microscope (Zeiss Axioscope A1). The condition of ice contamination was examined using bright-field microscopy, and the cell growth condition was examined using fluorescence microscopy. After the frozen samples examination, the vacuum load-lock system shipped selected sample grids to the TPS 24A1 endstation. The cryo-sample grids were maintained under cryogenic conditions during the shipping.

Cryo-SXT tilt series were obtained by using the full-field transmission soft X-ray microscope at the National Synchrotron Radiation Research Center at the TPS 24A1 beamline. The selected cell in the (ROI was imaged by soft X-ray at a photon energy of 520 eV. Tilt images at ROI were collected by rotating the sample from −68° to + 68° with a 1° step, and stack images were obtained by background correction. The image reconstruction and segmentation of stack images were performed by the freely available IMOD (https://bio3d.colorado.edu/imod/) software using filtered back projection (FBP) and iterative reconstruction algorithms.

### CRediT authorship contribution statement

**Chia-Chun Hsieh:** Writing – review & editing, Writing – original draft, Visualization, Methodology, Investigation, Formal analysis, Data curation. **Zi-Jing Lin:** Writing – review & editing, Methodology, Data curation. **Lee-Jene Lai:** Writing – review & editing, Visualization, Validation, Supervision, Resources, Methodology, Investigation, Funding acquisition, Conceptualization.

## Declaration of competing interest

The authors declare that they have no known competing financial interests or personal relationships that could have appeared to influence the work reported in this paper.

## Data Availability

The data that has been used is confidential.
